# Supplementary material for: Identification of Lifestyle Behaviors Associated with Recurrence and Survival in Colorectal Cancer Patients Using Random Survival Forests
Source: Cancers (Basel). 2021 May 18;13(10):2442. doi: 10.3390/cancers13102442 (PMC8157840; doi:10.3390/cancers13102442)
Supplement: Supplementary file 1 [file cancers-13-02442-s001.zip › cancers-1183637-supplementary.pdf]

cancers

MDPI

Supplementary Materials

Identification of Lifestyle Behaviors Associated with Recurrence and Survival in Colorectal Cancer Patients Using Random Survival Forests

Moniek van Zutphen, Fränzel J. B. van Duijnhoven, Evertine Wesselink, Ruud W. M. Schrauwen, Ewout A. Kouwenhoven, Henk K. van Halteren, Johannes H. W. de Wilt, Renate M. Winkels, Dieuwertje E. Kok and Hendriek C. Boshuizen

Table S1. Overview of all post-diagnosis lifestyle variables included in the Random Survival Forest models.

Lifestyle Variables	Description
**Diet (g/day)**	
Fruits & vegetables	Any type of fruits and vegetables
Fruits	Any type of fruits
Vegetables	Any type of vegetables
Legumes	Any type of legumes
Ratio of wholegrains to refined grains	
Wholegrains	Brown and wholemeal bread, rye bread, oats, wholemeal pasta, brown rice
Refined grains	White bread, croissant, cornflakes, muesli, white pasta, white rice
Fast foods, snacks, and sweets	French fries, crisps, pastry and biscuits, savoury snacks, candy, sauces, pizza, pancakes, sandwich fillings high in sugar of fat, refined grains, sweet dairy desserts, and diet soft drinks
Red and processed meat	Any type of red and processed meat
Red meat	Steak, organ meat, beef roll, pork tenderloin, minced meat
Processed meat	Sausages, bacon, ribs, ham, cold cuts
Poultry	Any type of poultry
Fish	Any type of fish, with a maximum of 4g lean fish[1]
Eggs	Boiled and fried eggs
Soy products	Soy, bean curd, soy milk, soy drink, soy yoghurt
Dairy	Milk, yoghurt, quark, sweet dairy desserts, sweetened dairy drinks, coffee creamer, and a maximum of 40g cheese[1]
Cheese	Any type of cheese
Sugary drinks	Any type of sugary drinks including fruit juice and sweetened dairy
Tea	Any type of tea
Herbal tea	Any type of herbal tea
Coffee	Any type of coffee
Mineral water	Any type of mineral water
Alcohol-free beer	Any type of alcohol-free beer
Ethanol from alcoholic drinks	Ethanol from beer, wine, liquor
Beer	Any type of alcoholic beers

Wine	Any type of wines
Liquor	Any type of liquor
Ratio of liquid fats to solid fats	
Solid cooking fats	Butter, low-fat butter, hard margarine, solid backing/frying fat, lard
Liquid fats & oils	Soft margarine, low fat spreads, liquid cooking fats, olive oil
Potatoes	Boiled or baked potatoes
Soups	Any type of soups
Energy intake (kcal/day)	Total intake based on all FFQ items
Dietary fiber	Total intake based on all FFQ items
Protein	Total intake based on all FFQ items
Plant protein	Total intake based on all FFQ items
Animal protein	Total intake based on all FFQ items
Total fat	Total intake based on all FFQ items
Saturated fat	Total intake based on all FFQ items
Monounsaturated fat	Total intake based on all FFQ items
Polyunsaturated fat	Total intake based on all FFQ items
Trans fat	Total intake based on all FFQ items
Carbohydrates	Total intake based on all FFQ items
Mono- and disaccharides	Total intake based on all FFQ items
Polysaccharides	Total intake based on all FFQ items
**Physical activity (min/week)**	
Moderate-to-vigorous activities	All activities ≥3 MET
Walking	Leisure time and commuting
Biking	Leisure time and commuting
Gardening	≥3 MET
Sports	All sports ≥3 MET
Vigorous household activities and odd jobs	≥3 MET
**Adiposity**	
Body mass index (kg/m^2)	Based on self-reported height and weight
Waist circumference (cm)	Difference from 94cm (M) or 80cm (F)
Waist hip ratio (cm)	Difference from 0.95 (M) or 0.80 (F)
**Smoking**	
Current smoker (yes/no)	
Number smoked per day	

[1]Based on the Dutch Healthy diet index {Looman, 2017 #3527}

Abbreviations: MET, metabolic metabolic equivalent value; M, male; F, female.

Table S2. Variables predictive of recurrence or all-cause mortality, based on variable importance, by tumor location.

**Variables Predictive of Recurrence**	**Colon** Number of times selected as predictive variable in 10 repetitions of RSF model	**Rectum** Number of times selected as predictive variable in 10 repetitions of RSF model
Stage	10	10
Adjuvant chemotherapy	10	10
Sugary drinks	10	0
Alcohol-free beer	10	5
Liquor	9	0
Trans-fats	8	1
Saturated fat	8	10
Fast foods	7	0
Soy products	0	10
Total fat	1	10
Wholegrains (percentage)	0	8
Dairy	6	0
Polyunsaturated fat	4	0
Alcohol	2	0
Physical activity	2	0
Tea	0	6
Age	0	5
Plant protein	0	3
Mono- and disaccharides	0	3
Household activities	0	2
**Variables predictive of all-cause mortality**		
Age	10	10
Stage	10	0
ASA classification	10	0
Fruit	10	0
Liquid fat & oil	10	0
Soup	0	10
Sugary drinks	0	10
Red meat	0	10
Processed meat	0	10
Animal protein	0	10
Chemotherapy	1	9
Fruit & vegetables	9	0
Polyunsaturated fat	2	7
Fish	0	5
Coffee	4	0

Liquor	4	0
Saturated fat	0	3
Potatoes	1	3
Dairy	3	0
Herbal tea	2	0
Plant protein	0	2
Beer	0	2
Education	0	2

Variables printed in *italics* are background variables, all other variables are lifestyle variables. Variables were selected as predictive based on their VIMP values. Only variables selected in ≥2 model repetitions are included in this table.
